# Potential Roles of 1-Aminocyclopropane-1-carboxylic Acid Synthase Genes in the Response of *Gossypium* Species to Abiotic Stress by Genome-Wide Identification and Expression Analysis

**DOI:** 10.3390/plants11111524

**Published:** 2022-06-06

**Authors:** Jie Li, Xianyan Zou, Guoquan Chen, Yongming Meng, Qi Ma, Quanjia Chen, Zhi Wang, Fuguang Li

**Affiliations:** 1 Xinjiang Research Base, State Key Laboratory of Cotton Biology, Xinjiang Agricultural University, Urumqi 830052, China; maggieli1106@126.com (J.L.); chqjia@126.com (Q.C.); 2 State Key Laboratory of Cotton Biology, Institute of Cotton Research, Chinese Academy of Agricultural Sciences, Anyang 455000, China; 13247299145@163.com (X.Z.); wangzhi01@caas.cn (Z.W.); 3 Zhengzhou Research Base, State Key Laboratory of Cotton Biology, School of Agricultural Sciences, Zhengzhou University, Zhengzhou 450001, China; cgq2019zms@163.com; 4 Western Agricultural Research Center, Chinese Academy of Agricultural Sciences, Changji 831100, China; mengyongming2022@163.com; 5 Key Laboratory of China Northwestern Inland Region, Ministry of Agriculture and Rural Affairs, Cotton Research Institute of Xinjiang Academy of Agricultural and Reclamation Science, Shihezi 832003, China; qmacotton@163.com

**Keywords:** *Gossypium hirsutum*, *ACS* genes, expression patterns, abiotic stress, ethylene

## Abstract

Ethylene plays a pivotal role in plant stress resistance and 1-aminocyclopropane-1-carboxylic acid synthase (ACS) is the rate-limiting enzyme in ethylene biosynthesis. Upland cotton (*Gossypium hirsutum* L.) is the most important natural fiber crop, but the function of ACS in response to abiotic stress has rarely been reported in this plant. We identified 18 *GaACS*, 18 *GrACS*, and 35 *GhACS* genes in *Gossypium* *arboreum*, *Gossypium* *raimondii* and *Gossypium* *hirsutum*, respectively, that were classified as types I, II, III, or IV. Collinearity analysis showed that the *GhACS* genes were expanded from diploid cotton by the whole-genome-duplication. Multiple alignments showed that the C-terminals of the GhACS proteins were conserved, whereas the N-terminals of GhACS10 and GhACS12 were different from the N-terminals of AtACS10 and AtACS12, probably diverging during evolution. Most type II *ACS* genes were hardly expressed, whereas *GhACS10*/*GhACS12* were expressed in many tissues and in response to abiotic stress; for example, they were highly and hardly expressed at the early stages of cold and heat exposure, respectively. The *GhACS* genes showed different expression profiles in response to cold, heat, drought, and salt stress by quantitative PCR analysis, which indicate the potential roles of them when encountering the various adverse conditions, and provide insights into GhACS functions in cotton’s adaptation to abiotic stress.

## 1. Introduction

The growth and development of cotton as well as other plants are regulated by phytohormones, such as jasmonic acid [1,2], ethylene [3,4,5,6,7], indole-3-acetic acid [8,9,10], and gibberellin [11,12,13]. Ethylene is an important and unique gaseous phytohormone that regulates various physiological processes including seed germination, seedling growth, leaf and flower senescence and abscission, photo-morphogenesis, and fruit ripening [14]. In cotton, exogenous ethylene induces leaf abscission in seedlings [15,16], causes alterations of radicle cells [17], and promotes fiber elongation [4]. *Gossypium* *arboreum* is known to produce short fiber and *Gossypium raimondii* has a fiberless phenotype [18]. During fiber development, these two species produce significantly lower and higher levels of ethylene, respectively, than upland cotton (*Gossypium* *hirsutum*), the cultivated plant that produces the long and white fiber that is used in the textile industry. Therefore, precise regulation of ethylene biosynthesis is crucial for cotton plant growth and fiber development.

In the Yang cycle, ethylene biosynthesis is completed in two steps: (1) the substrate S-adenosyl methionine is converted to the ethylene precursor 1-aminocyclopropane-1-carboxylic acid (ACC) and 5′-methylthioadenosine by ACC synthase (ACS); and (2) the substrate ACC is converted to ethylene, CO_2_, and cyanide by ACC oxidase [19,20]. Step 1, which involves ACS, is usually regarded as the rate-limiting step in ethylene biosynthesis [21], implying that ACS is the important enzyme in this pathway. The *Arabidopsis* genome encodes nine ACS enzymes with catalytic activity, two ACS enzymes with aminotransferase activity (AtACS10 and AtACS12), and a pseudogene (*AtACS3*) [22,23,24]. The nine ACS proteins contain a catalytic domain and were classified as type I, II, or III based on their structure: type I (AtACS1, AtACS2, AtACS6) contains target sites for mitogen-activated protein kinase (MAPK) and calcium-dependent protein kinases (CDPK); type II (AtACS4, 5, 8, 9, 11) contains CDPK and E3 ligase target sites at the C-terminal [23,24]; and type III (AtACS7) has no target site at the C-terminal but can be degraded by the interaction between its N-terminal and E3 ubiquitin-protein ligase XBAT32 [25,26] or protein phosphatases 2C [27]. By contrast, the interaction between ACS and 14-3-3 protein repressed the ubiquitination reaction to maintain the stability of the ACS proteins [28,29]. AtACS2, 6, 7, 8, 11 were shown to participate in pathogen invasion [30]; AtACS7, 9, 11 were reported to maintain homeostasis between ethylene, reactive oxygen species, and the brassinosteroid phytohormones [31]; and AtACS2, 5 were involved in abscisic acid-responsive pathways to regulate plant growth and development [32]. By contrast, AtACS4, 8 were negatively modulated by abscisic acid to reduce ethylene production [33]. ACS5 is stabilized by phosphorylation and ubiquitylation, and these post-translational modifications affected the ethylene biosynthesis pathway, which resulted in the change of ethylene production, thus regulated plant growth and development [26,29,34,35,36]. The ethylene-overproducing mutants *eto2* and *eto3* of *AtACS5* and *AtACS9*, respectively, result in the missense of the C-terminals of the proteins, and exhibit loss of enzymatic activity, indicating that the conserved C-terminal of the type II AtACSs is critical for AtACS activity and the regulation of ethylene biosynthesis [37,38]. In rice, *Os-ACS1*, *OS-ACS2*, and *Os-ACS3* could be induced by anaerobiosis and IAA [39] and the Os-ACS5 participated in root-shoot communication during submergence stress and might play a role under low-oxygen stress [40]. In cucumber, the *CS-ACS1G* is linked to the F locus and plays a pivotal role in the determination of sex in cucumber flowers [41,42]. In pear, the expression *PpACS1* was up-regulated under salicylic acid and IAA stress in fruit, indicating that PpACS1 might be involved in fruit ripening and response to SA [43]. In banana, the phosphorylation of serine 476 and 479 residues at the C-terminal region of MA-ACS1 is a key regulatory protein in banana fruit repening [44].

Members of the ACS multigene family are known to play pivotal roles in regulating ethylene biosynthesis, thereby affecting cotton plant growth, especially fiber development [4,18]. In *Arabidopsis*, *ACS* genes have been shown to be involved in the response to phytohormones signaling (e.g., abscisic acid), abiotic stresses, pathogens infection, and reactive oxygen species stimulus; however, few studies have reported the response of *ACS* genes to abiotic stress in cotton. In this study, we identified *ACS* genes in upland cotton and analyzed their expression patterns as well as the physicochemical properties and conserved domains of the encoded proteins under different abiotic stresses to discover the functional divergence of the *ACS* genes in response to different abiotic stresses.

## 2. Results

### 2.1. Identification and Phylogeny Analysis of the ACS Gene Family in Cotton

In total, 71 *ACS* genes were identified in cotton, including 35 in *G.* *hirsutum* (*GhACS*), 18 in *G.* *arboreum* (*GaACS*) and 18 in *G.* *raimondii* (*GrACS*). The lengths of the 35 *GhACS* genes in the genome ranged from 1512 bp (*Gh_DACS7.1*) to 3254 bp (*Gh_AACS2*), and the lengths of the protein-coding sequences ranged from 1290 bp (*Gh_AACS11.1*) to 1632 bp (*Gh_AACS10.2*). The number of amino acids in the encoded GhACS proteins ranged from 429 (Gh_AACS11.1) to 543 (Gh_AACS5), the molecular weight ranged from 48.32 kDa (Gh_AACS11.1) to 59.704 kDa (Gh_DACS10.1), and the isoelectric points (pI) ranged from 6.381 (Gh_AACS6.1) to 8.957 (Gh_DACS10.2) (Table S1).

**Figure 1:**
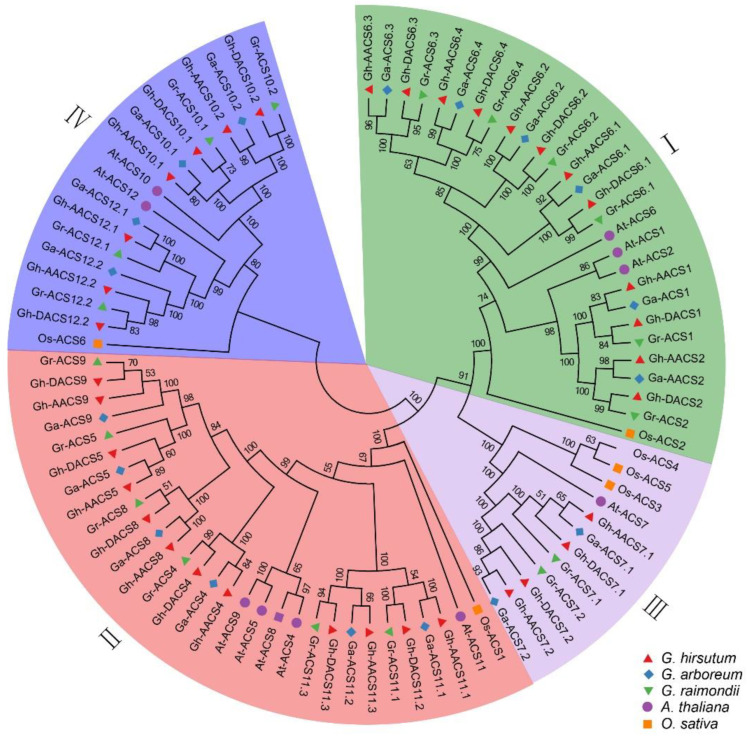
Phylogeny relationship of the ACS proteins in cotton and other species. The neighbor-joining phylogenetic tree was constructed based on a multiple sequences alignment of 88 ACS protein sequences from five species including *G. hirsutum* (GhACS), *G. arboreum* (GaACS), *G. raimondii* (GrACS), *Oryza sativa* (OsACS), and *A. thaliana* (AtACS), with 1000 bootstraps and model of *p*-distance, in which the ACS proteins family was divided into four subgroups. The different colored shapes: red triangle, blue diamond, green inverted triangle, purple circle, and orange square were used to indicate *G. hirsutum*, *G. arboreum*, G. raimondii, *A. thaliana*, and *O. sativa*, respectively.

To explore the phylogenetic relationship among the cotton *ACS* genes, 88 ACS protein sequences from cotton, *Arabidopsis*, and rice were used to construct a phylogenetic tree. As shown in Figure 1, the 88 ACS proteins clustered into four types. Among the cotton ACS proteins, the type I group contained two GhACS1, two GhACS2, and eight GhACS6 proteins; the type II groups contained two GhACS4, two GhACS5, two GhACS8, two GhACS9, and four GhACS11 proteins; the type III group contained four GhACS7; and the type IV group contained four GhACS10 and three GhACS12 proteins (Figure 1). AtACS10 and AtACS12, which were included in the type IV group, have been reported to have no ACS catalytic activity [23].

### 2.2. Analysis of the C-Terminal of the ACS Proteins

**Figure 2:**
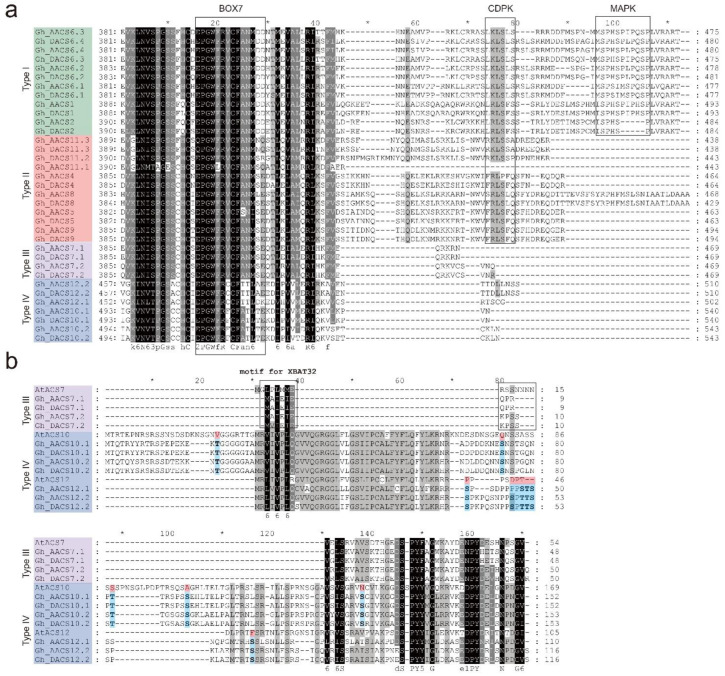
Comparison of the N-terminal and C-terminal domains in ACS proteins: (**a**) Multiple sequences alignment of GhACS proteins by ClustalW. The C-terminal sequences are shown. The rectangles indicate the conserved BOX7, CDPK, and MAPK motifs. (**b**) Multiple sequences alignment of the type III and IV GhACS proteins by ClustalW. The N-terminal sequences are shown. The black boxes indicate the conserved short XBAT32 regions. Possible phosphorylation sites (Ser/Thr) are in bold font. Amino acid differences between the GhACS and AtACS proteins are highlighted using different colors.

In a previous study, the AtACS proteins were classified as types I–III based on the C-terminal sequence, and the key roles of the ACS C-terminal in function differentiation were recognized [23]. Here, the multiple sequence alignment showed that the GhACS proteins were similar to the AtACS proteins within each type of ACS proteins. All the GhACS proteins contained the catalytic domain (amino acids 26–418 in Gh_AACS1), and the conserved BOX7 sequence in the catalytic domain as shown in Figure 2a. The 12 type I GhACS proteins contained the CDPK and MAPK sites, and the type II GhACS proteins contained only the CDPK motif, except for Gh_AACS11.1, which had no CDPK motif suggesting it may have lost its catalytic function during evolution. Among the two type III ACS proteins, GhACS7 contained a short region that could interact with XBAT32 (Figure 2b) that was similar to the AtACS7 region; the slight difference may be the result of evolution. The type III and IV ACS protein alignment showed that there was an additional long region in the N-terminal of the ACS10 proteins, and several serine/threonine sites in GhACS10 and GhACS12 were different from those in AtACS10 and AtACS12, respectively (Figure 2b).

### 2.3. Analysis of Chromosomal Distribution, Gene Structure, and Motifs

**Figure 3:**
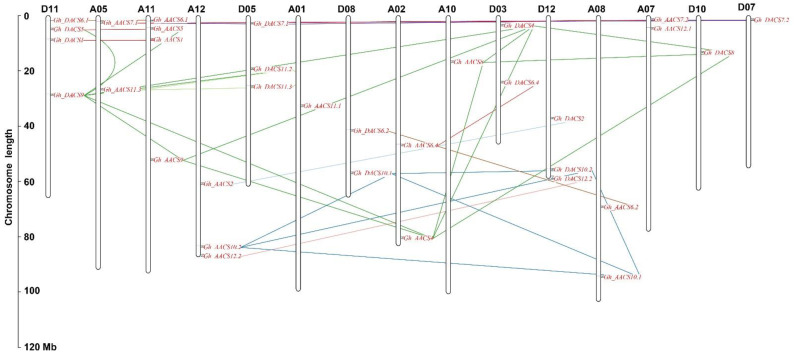
Chromosomal localization of the *GhACS* genes. Thirty-three of the 35 *GhACS* genes were localized to upland cotton chromosome regions; the other two, *Gh_AACS6.3* and *Gh_DACS6.3*, were localized to scaffolds. Lines indicate segmental duplications of the *GhACS* genes. Genes linked by same color line are paralogous genes pairs.

We identified 17 and 16 *GhACS* genes on the eight chromosomes of At-subgenome and seven chromosomes of Dt-subgenome, respectively (Figure 3); the remaining two *GhACS* genes (*Gh_AACS6.3* and *Gh_DACS6.3*) were localized to scaffold3404_A12 and scaffold4588_D12 and are not shown in the figure. The *GhACS* genes were localized on chromosomes A01, A02, A05, A07, A08, A10, A11, A12, D03, D05, D07, D08, D10, D11, and D12.

**Figure 4:**
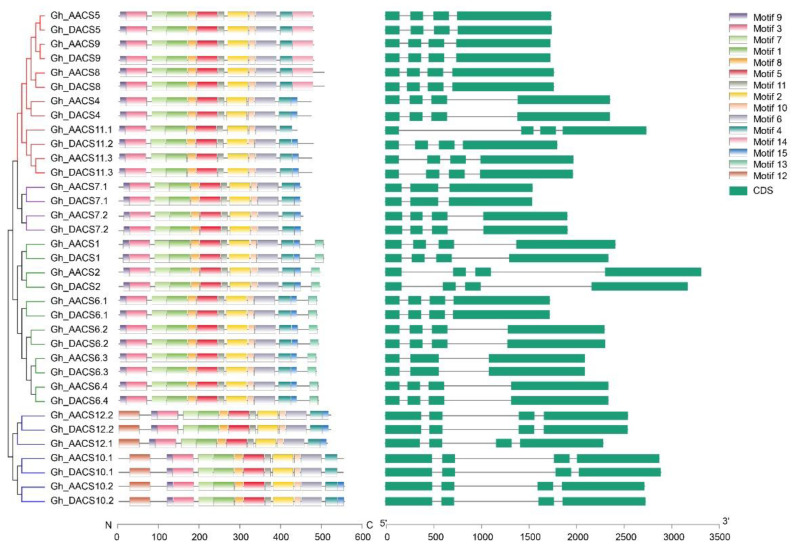
Conserved motifs in the GhACS proteins and exon–intron structure of the *GhACS* genes.

The similar exon–intron structure of the *GhACS* genes suggests that they belong to the same sub-branch in the phylogenetic tree (Figure 4). The numbers of exons and introns in the *GhACS* genes were similar; 88.6% (31 of 35) *GhACS* genes had four exons and three introns. The exceptions were *Gh_AACS7.1*/*Gh_DACS7.1* and *Gh_AACS6.3*/*Gh_DACS6.3*, which contained only three exons. In all the *GhACS* genes, the exons closest to the 3′ ends were longer than the exons at the 5′ ends, but the lengths of the introns were significantly different. For example, the third intron of *Gh_AACS2* (1173 bp) was 1090 bp longer than the third intron of *Gh_AACS5* (83 bp).

Conserved motifs were identified in the GhACS protein sequences and analyzed using HMMScan (https://www.ebi.ac.uk/Tools/hmmer/search/hmmscan, accessed on 6 April 2022) (Figure 4, Table S2). Among them, motifs 1, 2, and 4–7 were aminotransferase classes I and II, motif 3 was the BRK domain, and motif 12 was Fanconi anemia-associated. All the GhACS proteins contained motifs 1–11; GhACS5, GhACS8, and GhACS9 contained a unique motif 14; the type I GhACS proteins contained motif 13; and the type IV GhACS proteins contained motif 12. The differences in the motifs may contribute to the divergence in function of the GhACS proteins.

### 2.4. Collinearity and Ka:Ks Analysis

**Figure 5:**
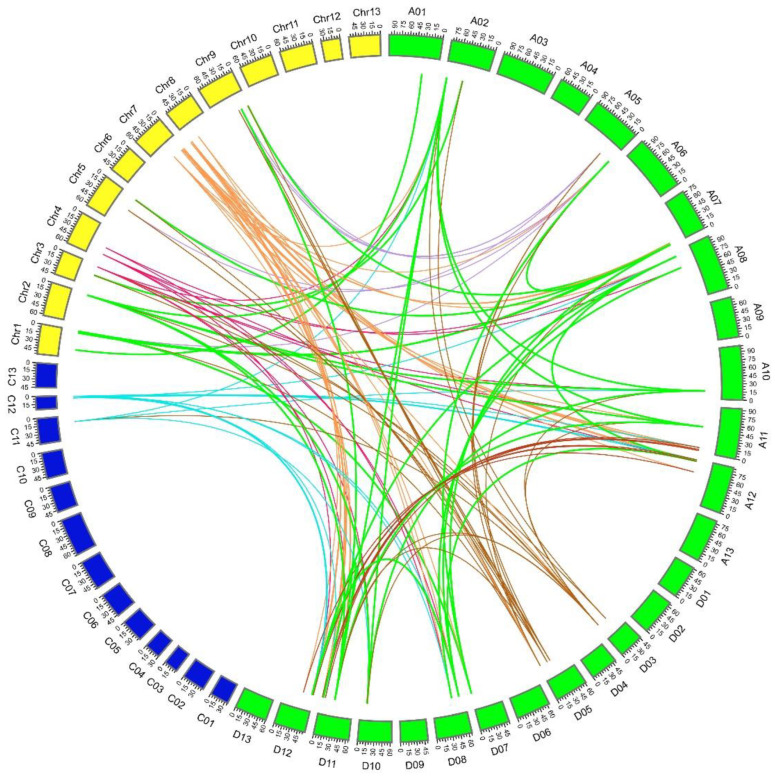
Circos map of 134 homologous *ACS* gene pairs among *G. arboreum*, *G. raimondii*, and *G. hirsutum*.

**Figure 6:**
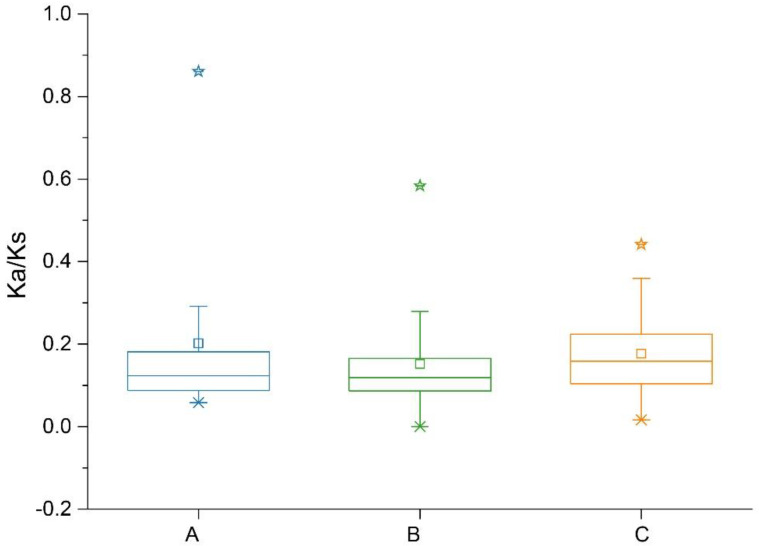
Distribution of Ka:Ks ratios between homologous gene pairs among *G. arboreum*, *G. raimondii*, and *G. hirsutum*. The Ka:Ks ratios of *ACS* gene pairs between *G. arboreum* and *G. hirsutum* (abscissa A), between *G. raimondii* and *G. hirsutum* (abscissa B), and in the *G. hirsutum* genome (abscissa C) are shown. The pentagrams, suqares, asterisk and long lines in boxes represented the outliers, means, minimum vaules and median line in each group of the Ka:Ks ratios, respectively; Boxes represented the 25%~75% range of the Ka:Ks ratios; The two short lines up and down represent range within 1.5 interquatile range.

*G. hirsutum* originated from a whole-genome duplication event experienced by *G. arboreum* and *G. raimondii* approximately 1–2 million years ago [45], which led to the formation of *GhACS* gene family. The collinearity analysis detected 50 and 49 pairs of orthologous genes between *G. hirsutum* and *G. arboreum* and between *G. hirsutum* and *G. raimondii*, respectively, and 35 pairs of paralogous genes were also identified in the upland cotton genome (Figure 5, Table S3). The whole-genome duplication analysis showed that the *GhACS* genes were expanded by segmental duplication. The paralogous gene pairs are linked with different color lines in Figure 3. No tandem duplications were detected between the *GhACS* genes. The Ka:Ks ratios (ratio of the number of nonsynonymous substitutions per nonsynonymous site to the number of synonymous substitutions per synonymous site) of all gene duplication were also calculated. The Ka:Ks ratios ranged from 0.058 to 0.861 between *GhACS* and *GaACS*, 0 to 0.584 between *GhACS* and *GrACS*, and 0.016 to 0.442 for the paralogous genes on the *G. hirsutum* At and Dt chromosomes. Overall, the Ka:Ks ratios were <1.0 (Figure 6, Table S2).

### 2.5. Analysis of GhACS Expression Patterns in Tissues and in Plants under Stress

**Figure 7:**
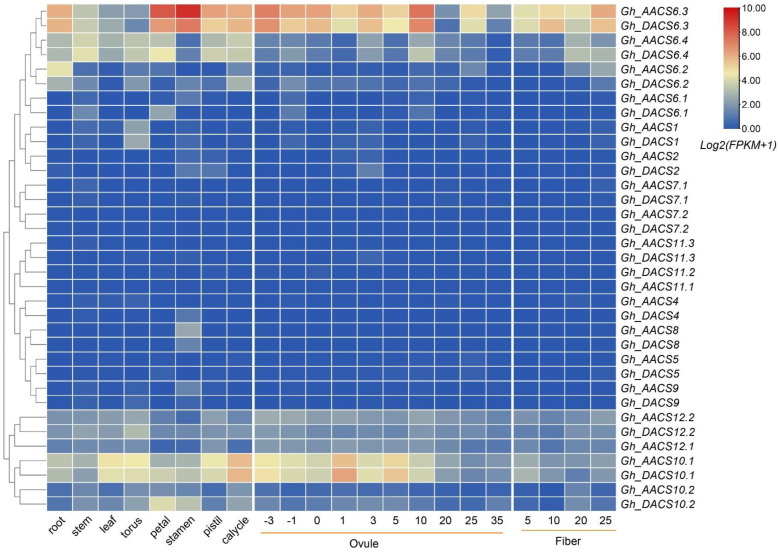
Tissue-specific expression patterns of the *GhACS* genes. The heatmap shows the expression levels of *GhACS* genes in 22 tissues, including root, stem, leaf, torus, petal, stamen, pistil, calycle, ovules, and fibers, at different development stages.

To characterize the *ACS* expression patterns, we downloaded and re-analyzed RNA-Seq data from NCBI’s SRA database. The heatmap showed that the type I and IV *GhACS* genes were expressed in most of the tested tissues (Figure 7) and that *Gh_AACS1*/*Gh_DACS1* and *Gh_AACS8*/*Gh_DACS8* were specifically expressed in torus and stamen, respectively. Notably, *Gh_AACS6.3*/*Gh_DACS6.3* and *Gh_AACS10*/*Gh_DACS10* were expressed in all the tissues, suggesting that they may play key roles in the cotton plants’ growth and fiber development, even though *ACS6* is not considered as fiber preferential [4]. For other *ACS* genes, such as the type II *GhACS4*, *GhACS5*, *GhACS8*, *GhACS9*, and *GhACS11* and type III *GhACS7* genes, very low or no transcription was detected in the tissues (Figure 7).

**Figure 8:**
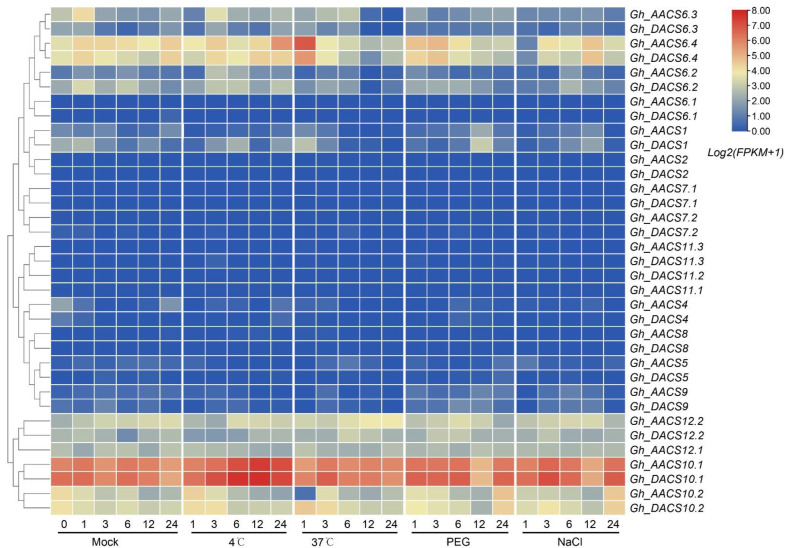
Expression patterns of the *GhACS* genes in response to abiotic stresses. The heatmap shows the expression changes of *ACS* genes in *G*. *hirsutum* under cold, hot, drought, and salt stress at different exposure times.

Because the *ACS* genes encode enzymes in the ethylene biosynthesis pathway, and because ethylene plays critical roles in abiotic stress [46,47,48], we used RNA-Seq data to analyze the expression patterns of the *GhACS* genes in response to cold, heat, salt, and drought. Similar to the findings for *GhACS* genes in tissues, the type I and IV *ACS* genes responded to all four abiotic stresses (Figure 8). When the seedlings were exposed to high temperature (37 °C) for 1 h, the expression of *Gh_AACS6.4*/*Gh_DACS6.4* and *Gh_AACS10.2* was quickly up- and down-regulated, respectively, and then remained stable, suggesting that these *ACS* genes responded positively and negatively at the early stage (1 h) of heat stress. *Gh_AACS6.4*/*Gh_DACS6.4* also responded to drought when exposed for 1–3 h, and to salt stress when exposed for 12 h. The expression of *Gh_AACS10.1*/*Gh_DACS10.1* was up-regulated when exposed to low temperature (4 °C) for 6–24 h, indicating that they responded to cold stress. These results suggest that the *GhACS* genes have functional divergence when the plants are exposed to abiotic stress.

### 2.6. Analysis of GhACS Genes Expression Patterns in Plants under Abiotic Stress by Quantitative PCR (qPCR)

**Figure 9:**
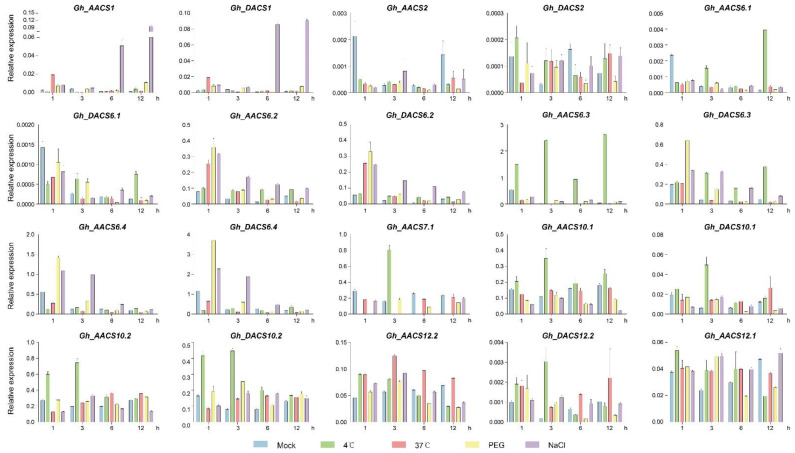
Expression levels of *GhACS* genes under cold, heat, drought, and salt stresses at different times by quantitative PCR analysis.

We performed qPCR analysis to validate the expression patterns of *ACS* genes in plants under cold (4 °C), hot (37 °C), drought (polyethylene glycol, PEG), and salt (NaCl 400 mM) stress for 0, 1, 3, 6, and 12 h. *Gh_AACS1*/*Gh_DACS1* had similar expression patterns and were up-regulated in response to salt stress after 6–12 h exposure and to heat stress after 1 h exposure, whereas *Gh_AACS2*, *Gh_DACS6.1*, *Gh_AACS6.2*, *Gh_DACS6.2*, *Gh_AACS6.4*, and *Gh_DACS6.4* were down-regulated in response to salt stress after 6–12 h exposure (Figure 9). Most of the *GhACS6* genes responded to PEG-induced drought stress at the early stage, the exception was *Gh_AACS6.1*. The expression of *Gh_AACS10.2*, *Gh_DACS10.2*, and *Gh_AACS12.1* did not change in response to drought. *Gh_AACS6.1*/*Gh_DACS6.1* and *Gh_AACS6.3*/*Gh_DACS6.3* responded to cold stress after 12 h exposure, and *Gh_AACS7.1*, *Gh_AACS10.1*, *Gh_DACS10.1*, *Gh_AACS10.2*, *Gh_DACS10.2*, and *Gh_DACS12.2* responded to cold treatment after 1–3 h. Interestingly, *Gh_AACS10.2* and Gh_DACS10.2 showed high expression at the early stage of cold exposure and high expression at the early stage of heat stress. *Gh_AACS12.2* and *Gh_DACS12.2* both responded to cold and heat stress, whereas *Gh_AACS12.1* expression did not change in response to cold or heat stress. These results suggested that *GhACS* genes are involved in the response to a variety of abiotic stress conditions during cotton growth and development.

## 3. Discussion

ACSs are recognized as the rate-limiting enzymes in ethylene biosynthesis that determine ethylene accumulation and signal transduction in plant development and abiotic stress tolerance. The different roles of ACSs in response to different abiotic stress and diverse regulatory mechanisms have been widely reported [27,29,46,47,48,49]. Here, we identified 35 *ACS* genes from cultivated cotton *G. hirsutum*. The numbers of *ACS* genes in tetraploid cotton are approximately double the numbers in diploid cotton, indicating that the *ACS* genes were expanded only through whole-genome-duplication [18]. However, the numbers of *ACS* genes in a monocot (six in rice) and in a dicot (nine in *Arabidopsis*) imply that *ACS* genes may have undergone gene division from angiosperms [50]. In *Arabidopsis*, AtACS10 and AtACS12 were reported to have no catalytic activity [23], and their function is largely unknown. However, *GhACS10/GhACS12*, the homologous genes of *AtACS10/AtACS12*, seem to play roles in cotton plant growth and response to abiotic stress because they were expressed in many tissues, including ovules, stamen, and calycle, and their expression levels changed in response to abiotic stress. The multiple sequence alignment showed several possible phosphorylation sites on serine and threonine residues, which were different from those in the N-terminal of *Arabidopsis* ACSs, suggesting that GhACS10 and GhACS12 are type IV proteins that may have divergent functions. These findings suggest that the unique ethylene signaling controlled by ACSs seems to occur at different developmental stages or under different environmental conditions and may play essential roles in plant evolution.

Ethylene is a fruit-ripening phytohormone that has multifaceted roles in plants. Ethylene that is synthesized in response to environmental stresses can either exacerbate the symptoms of stress or enhance plant survival depending on the species, age, and the type of stress (VanLoon and Glick 2004). The different effects of ethylene have been explained by the two-phase model proposed by Glick et al. (2007a), in which the ACS-mediated synthesis of additional ACC may be harmful to the plant by causing a second ethylene peak that could result in senescence, chlorosis, and abscission of the plant (Ciardi et al. 2000; VanLoon and Glick 2004). Ethylene usually participates in plants’ abiotic stress responses by regulating the accumulation of reactive oxygen species. In cotton, ethylene production plays key roles in fiber development [4] and the abiotic stress response [12,49,51]. In apple (*Malus domestica*), the ADP-ribosylation factor MdARF5 bound to the promoter of *MdACS1* and *MdACS3* to activate ethylene biosynthesis in apple fruit ripening [52]. In *Arabidopsis*, the type II ACS5 protein acts as a scaffold that links SINAT2 and EOL2 to form a functional complex and increase the stability of ACS5, which is critical for the autophagy stress response triggered by ethylene biosynthesis and brassinosteroid signaling [53]. These findings suggest that ACS may be a central molecular component that can transduce environmental signals into cells via crosstalk with phytohormone pathways. The expression pattern of the *GhACS* genes in RNA-Seq analysis is not consistent with those in QPCR, which might be attributed to the different genotypes of materials and the sampling methods. In detail, TM-1 and the leaf tissues were planted and sampled in the RNA-Seq experiments; however, the cultivar ZM24 was used in our study and the whole seedlings were sampled for the QPCR. Nevertheless, we found that the transcription of only a few of the *GhACS* genes changed in response to heat, cold, and osmotic (PEG and NaCl treatment) stresses. *GhACS1* was significantly up-regulated after exposure to NaCl for a long time, and *GhACS6.3*, *GhACS7.1*, and *GhACS10/12* showed different induction patterns in response to cold stress. These results indicate that the type I, III, and IV ACSs, but not the type II ACSs, may be involved in the tolerance of cotton to various abiotic stresses, which is different from the results reported for *Arabidopsis* ACSs. Serine and threonine are common phosphorylation sites and their phosphorylation can regulate protein activity and stability and is critical for protein function [54,55]. In the type IV ACS proteins, serine/threonine phosphorylation sites were identified in cotton but not in *Arabidopsis*, indicating that *GhACS10* and *GhASC12* may have different functions and regulatory mechanisms than the corresponding genes in *Arabidopsis*. Therefore, the different ACSs may mediate abiotic stresses in different plant species, indicating the functional diversity of ACSs in adapting to external conditions. However, how the cotton ACSs are regulated at both the transcriptional and translational levels is largely unknown, and therefore much effort is needed to explore this.

The ACS enzymes played pivotal roles in plant growth and response to abiotic stress. In this study, we identified *GhACS* genes in cotton that encoded proteins with diverse motif patterns that function in fiber development and abiotic stress tolerance. The results imply the complexity of ethylene synthesis and signaling in the cotton development and growth. Furthermore, the GhACSs that were found to be involved in different stress conditions may provide clues for further studies of ethylene function in abiotic stress tolerance of cotton.

## 4. Material and Methods

### 4.1. Identification of ACS Genes in Cotton

The protein sequences of *G. hirsutum*, *G. arboreum*, and *G. raimondii* were obtained from the CottonGen database (https://www.cottongen.org/, accessed on 3 March 2022), and the protein sequences of rice (*Oryza sativa*) were obtained from the Phytozome database (https://phytozome-next.jgi.doe.gov/, accessed on 3 March 2022). The sequences of the 12 *Arabidopsis* ACS proteins were downloaded from The Arabidopsis Information Resource v10 (TAIR10; https://www.arabidopsis.org, accessed on 3 March 2022) and used as the query sequences in BLAST searches (e-value of 1 × 10^−5^) against the cotton and rice protein sequences. The domain structure of all the protein sequences was determined using the HMMScan program in the HMMER 3.0 package [56]. Only sequences that contained an aminotransferase class I and II “Aminotran_1_2” (PF00155) domain were considered to be ACS proteins [56]. The taxa labels and gene IDs of the identified ACS proteins are listed in Table S1.

### 4.2. Phylogenetic Tree Construction

The amino acid sequences of the ACS proteins of *A. thaliana*, *Gossypium*, and *O. sativa* were aligned using the ClustalW program integrated in MEGA 6.06 (v 6.06) software [28]. A neighbor-joining phylogenetic tree was constructed based on the multiple sequence alignment, with the model of *p*-distance and the bootstrap as 1000 replicates.

### 4.3. Chromosomal Location, Gene Structure, and Motif Analysis

The physical positions of the upland cotton *ACS* genes on the chromosomes were obtained from the GFF3 file that we downloaded from the CottonGen database (https://www.cottongen.org/, accessed on 18 April 2022) [57] and visualized usingTBtools [58]. The exon–intron structure of the *GhACS* genes was visualized using the Gene Structure Display Server v2.0 [59]. The conserved motifs of the GhACS proteins were analyzed using the MEME suite (https://meme-suite.org/meme/, accessed on 6 March 2022) [60] with the following parameters: maximum number of motifs, 15; minimum motif width, 6; and maximum motif width, 50.

### 4.4. Plant Growth and Tissue Sampling

*G. hirsutum* cv. ZM24 seedlings were grown in Hoagland nutrient solution for 2 weeks [61]. Then, the seedlings were exposed to PEG-induced drought (20% PEG 6000) [62], cold (4 °C), heat (37 °C), or high salt (400 mM NaCl) conditions [49]. In detail, for the drought and salt treatment, PEG 6000 and NaCl were added into Hoagland nutrient solution with a final concentration of 20% (*w*/*v*) and 400 mM, respectively. For the cold and hot stress, the plants were put into 4 °C and 37 °C conditions. The seedlings were sampled at 0, 1, 3, 6, and 12 h, after the abiotic stress treatment, there were three biological replicates.

### 4.5. Expression Patterns Analysis

For the RNA-Seq data analysis, the raw transcriptome data were downloaded from NCBI’s SRA database (PRJNA490626) [63], and analyzed using the HISAT2-StringTie software [64,65,66]. The expression patterns were visualized using TBtools with a homogenize method of log_2_(FPKM+1) [58].

For the qPCR analysis, total RNA was extracted using the RNAprep Pure Kit (for polysaccharides and polyphenolics-rich plants) (Tiangen, Beijing, China). The RNA was quantified using a NanoDrop 2000 spectrophotometer (Thermo Fisher Scientific, USA), and the quality was determined by 1% agarose gel electrophoresis. The absorbance ratio A260/280 was 1.8–2.1. Approximately 1 μg RNA was used as the template to synthesize cDNA using TransScript II All-in-One First-Strand cDNA Synthesis SuperMix for qPCR (AT301-02, TransGen) according to the manufacturer’s instructions. The qPCR experiments were performed using a LightCycler 480 system with a SYBR-Green Real-time PCR SuperMix (AQ101-01, TransGen), with three biological and three technical replicates for each sample. The cotton histone 3 gene (*GhHistone3*; AF024716) was used as the internal gene. The 2^−∆^ Ct method was used to calculate the relative expression of each gene, with three technical repetitions and three biological repetitions [67]. Data are shown as mean ± SD. The Student’s t-test was used for the significance statistic. The primer sequences were designated using NCBI Primer-BLAST (https://blast.ncbi.nlm.nih.gov/, accessed on 10 March 2022) and are listed in Table S4.

## Data Availability

Not applicable.

## Supplementary Materials

The following supporting information can be downloaded at: https://www.mdpi.com/article/10.3390/plants11111524/s1, Table S1. Chromosomal locus ID and lengths of the *ACS* genes in cotton. Table S2. Details of the 15 conserved motifs in the GhACS proteins of *G. hirsutum*. Table S3. Selective pressure of homologous *ACS* genes among *G. arboreum*, *G. raimondii*, and *G. hirsutum*. Table S4. Primer sequences used in this study.
